# Optic nerve Hemangioblastoma with bilateral frontal lobe Oedema: a case report

**DOI:** 10.1186/s12886-020-01706-4

**Published:** 2020-11-03

**Authors:** Shujia Xu, Qian Li, Bingyang Bian, Hongli Zhou, Dan Li

**Affiliations:** 1 The First Hospital of Jilin University, Changchun, China; 2 The Second Hospital of Jilin University, Changchun, China

**Keywords:** Optic nerve, Hemangioblastoma, VHL syndrome

## Abstract

**Background:**

Hemangioblastomas are rare benign tumours that are most commonly detected in the subtentorium or spinal cord. Optic nerve hemangioblastoma is very rare and is most commonly associated with Von Hippel-Lindau (VHL) syndrome.

**Case presentation:**

Here, we report a case of hemangioblastoma of the optic nerve with bilateral frontal lobe oedema without VHL syndrome, which has not yet been reported. A 51-year-old woman presented with progressive and painless deteriorating vision in the left eye. Magnetic resonance imaging showed a mass at the back of the left orbital optic nerve. Endoscopic-assisted intraorbital tumour resection was performed successfully. The pathological diagnosis was left optic nerve hemangioblastoma.

**Conclusions:**

This is the first reported case of optic nerve hemangioblastoma (HBL) with bilateral frontal lobe oedema.

## Background

Hemangioblastomas (HBLs) are benign tumours of the central nervous system that are often associated with Von Hippel-Lindau (VHL) syndrome. The main ophthalmological manifestation is retinal HBL, and it is very rare for HBLs to occur in the optic nerve. At present, only 35 cases of optic nerve HBLs have been reported in the literature. In this report, we describe a case of HBL of the optic nerve with bilateral frontal lobe oedema, a previously unreported manifestation of optic nerve HBL.

## Case presentation

A 51-year-old female patient presented with progressive and painless deteriorating vision in the left eye for 1 month and was treated in April of 2018. Her past medical history included 10 years of hypertension and diabetes and 6 years of heart disease. Her family medical history was also recorded. After fundus examination, her symptoms were diagnosed as ischaemic optic neuropathy in the left eye. After vasodilation treatment, visual acuity decreased further, accompanied by left exophthalmos. The patient returned for treatment on June 8, 2018. The ophthalmological examination results were as follows: best corrected visual acuity was 20/20 in the right eye; finger counting/50 cm in the left eye; and exophthalmos was 13 mm in the right eye and 16 mm in the left eye. The orbital distance was 97 mm. The cornea was transparent in both eyes. The pupils were 3 mm, and were of the same size and roundness in both eyes with light reflex. The crystalline lens and vitreous body were nebulous. The fundus examination results were as follows: no abnormality in the right eye; the boundary of the optic disc in the left eye was not clear (instead, it was pale); and the cup/disc ratio was 0.9. The visual field (VF) results revealed no abnormality in the right VF, while only the inferior nasal VF remained in the left eye. A visual evoked potential (VEP) test showed no obvious abnormality in either eye at peak time. The amplitude of the p2 wave in the left eye was lower than that in the right eye.

**Fig. 1:**
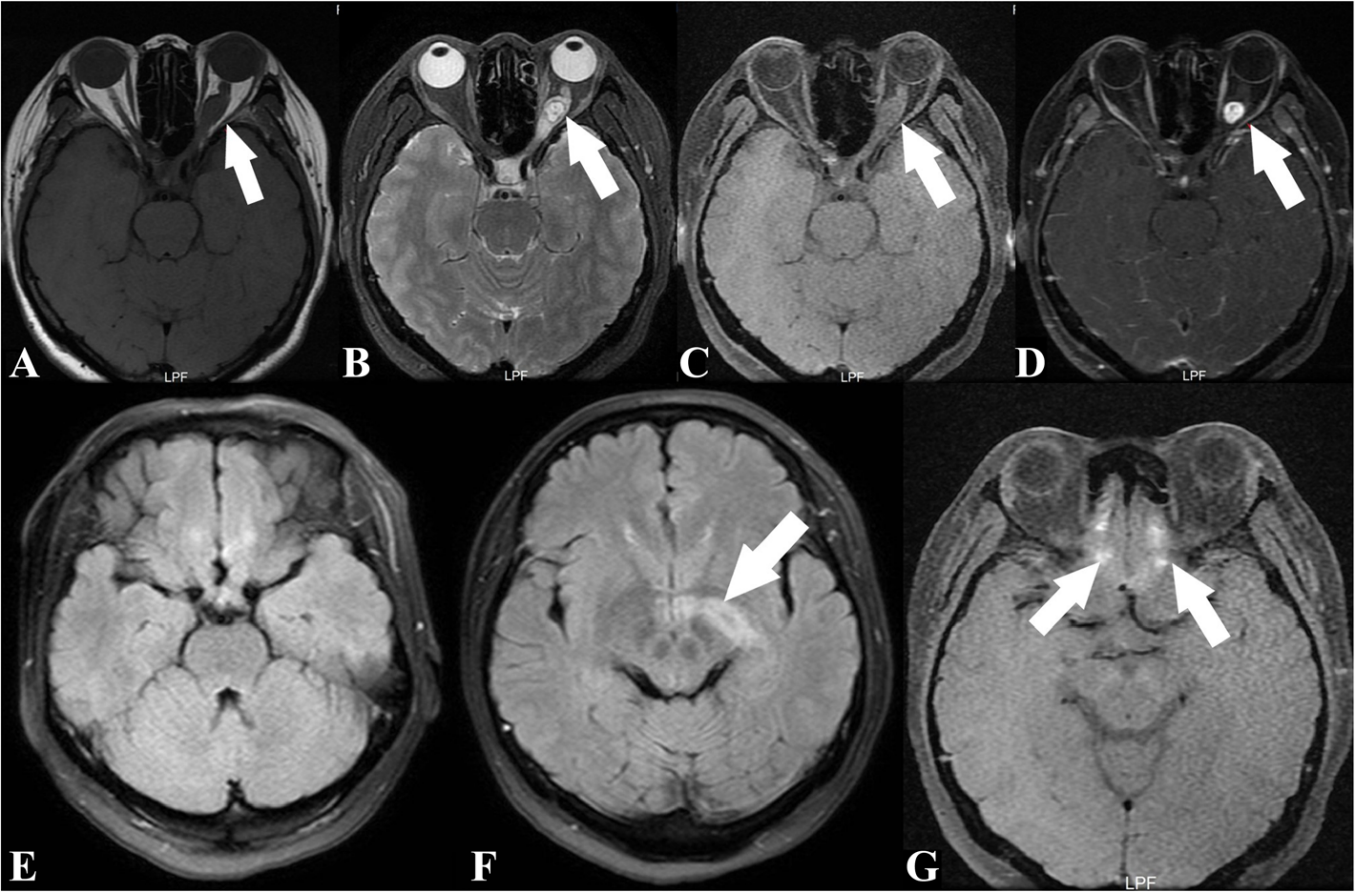
Comparison of preoperative and postoperative MRI. The T1- and T2-weighted images show optic neuropathy before the operation. The T1 image shows isointensity (**a**), and the T2 and FLAIR images show hyperintensity (**b**, **c**). There was a significantly enhanced signal in the tumour after the enhancement scan (**d**). The FLAIR image shows hyperintensity of the left optic chiasma, visual radiation, and bilateral frontal lobe (**e**, **f**, **g**)

Magnetic resonance imaging (MRI) showed a mass at the back of the left orbital optic nerve, with isointensity on T1 and hyperintensity on T2/fluid-attenuated inversion recovery (FLAIR) imaging. Heterogeneous enhancement was found on a contrast-enhanced scan (Fig. 1: a, b, c, d). There was no obvious flow void in or around the tumour. The proximal end of the optic nerve on the left side of the orbit was partially enlarged and tortuous, and the optic nerve sheath was dilated. There was no obvious abnormality in the right optic nerve. FLAIR imaging showed hyperintensity of the chiasma, left optic tract, and bilateral frontal lobes (Fig. 1: e, f, g).

Endoscopic-assisted intraorbital tumour resection was performed while the patient was under general anaesthesia, during which the medial orbital wall and the anterior part of the optic canal were incised from the nasal cavity. The optic nerve was cut off at the orbital apex, the surrounding tissues of the tumour were separated, and the tumour was completely removed by approaching from the nasal bulbar conjunctiva incision.

Pathological examination revealed that the tumour was mainly composed of abundant interstitial cells and capillaries. The cytoplasm of the interstitial cells was rich and lightly stained, showing vacuoles. Immunohistochemical staining showed positivity for CD34, CD31, and CD56 and negativity for progesterone receptor, creatine kinase (AE1/AE3), glial fibrillary acidic protein, S-100, epithelial membrane antigen, and D2–20. The pathological diagnosis was HBL of the left optic nerve.

**Fig. 2:**
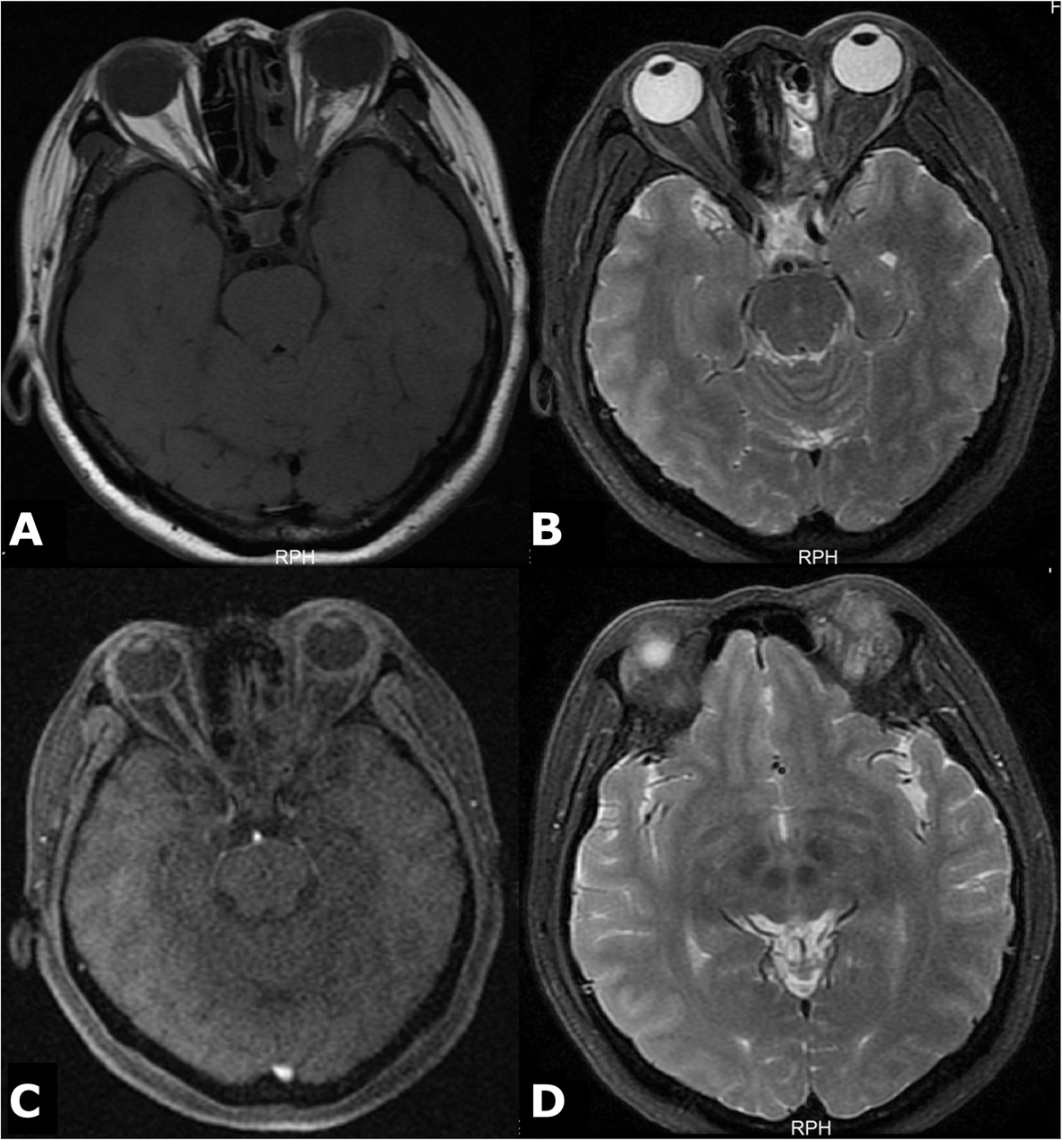
Three months after the surgery, T1-weighted images (**a**), T2-weighted images (**b**) and FLAIR images (**c**) all showed that the patient’s tumour had been excised and the signs of frontal lobe oedema had disappeared on T2-weighted images (**d**)

Three months after the operation, MRI showed that the tumour had been removed, and the signs of bilateral frontal lobe and optic choroid oedema had disappeared (Fig. 2).

## Discussion and conclusions

HBLs are benign tumours of the central nervous system that originate from the mesenchymal tissue surrounding the blood vessels and are remnants of mesodermal cells. HBLs account for only 1–2% of primary tumours in the central nervous system, and are most commonly found in the subtentorium or spinal cord [1, 2]. Clinically, HBLs are associated with VHL syndrome, an autosomal dominant genetic disease [3], with approximately 20% of patients having a family history. HBLs are more evident in men than in women, with a predominance of onset in young adulthood. HBLs are vascular tumours that are significantly enhanced during neuroimaging examination and are histologically characterised by a marginal mass of the vessel, generally of reddish colour. Microscopically, HBLs are characterised by vacuolar interstitial cells and abundant capillary networks. The main ocular complications of retinal HBLs are retinal exudation and tractional retinal detachment [4, 5].

The development of the disease is characterised by exophthalmos. The diagnosis of retrobulbar tumorus can be confirmed by neuroimaging, but the nature of the tumours is not clear. A total of 35 cases of optic nerve HBLs have been reported in the literature, 71% (25 cases) of which were associated with VHL syndrome [1]. Therefore, when diagnosing optic nerve HBLs, whole-body nerve tissues and organs should be examined to determine whether VHL syndrome also exists. As this patient had no family history of VHL or extraocular tumours, she was not diagnosed with VHL syndrome. However, the diagnosis of VHL syndrome could not be ruled out. It has been reported in the literature that there is a positive correlation between age and the risk of tumour development. Some patients may be diagnosed with VHL later in life and achieve gene penetrance at the age of 65 years [6]. Therefore, in our patient, who was 51 years of age, the diagnosis of VHL cannot be completely rejected. Genetic analysis can provide diagnostic evidence, but patients only sometimes have mutations in the gene [7].

At present, there is no effective treatment for the disease. Surgical resection of the tumour is currently the best method for treatment of optic nerve HBLs [7]. Turel et al. considered that early surgical treatment was needed when the tumour was far away from the optic disc and did not invade the optic nerve [8]. Comprehensive preoperative diagnosis, evaluation of the advantages and disadvantages of surgery, and effective communication with the patient are very important for the guidance of treatment.

The preoperative differential diagnoses of optic nerve HBLs includes optic nerve meningioma, optic nerve glioma and other conditions [3, 8, 9]. The aforementioned diseases are difficult to distinguish based on symptoms and physical examination results. In addition to a general history, neuroimaging examination can provide certain diagnostic data. The most sensitive methods for the examination of optic nerve HBLs are noncontrast MRI and contrast MRI. One of the remarkable features of these tumours is the large amount of oedema around the tumour, which can extend from the optic nerve to the optic chiasma, and optic radiation. It can even involve bilateral optic nerves. Typical HBLs have a significantly enhanced nodule on the wall of the unenhanced cyst. Larger HBLs usually have visible irregular flow cavities adjacent to the nodules, but these are rarely seen on the smaller HBLs [7]. In addition, the oedema of surrounding tissue caused by the tumour and the blood vessel flow void can be clearly observed on T2-weighted images, which strongly suggests the existence of HBL [1].

In this case, the MRI of the left optic neuropathy showed isointensity on T1 and hyperintensity on T2/FLAIR. After contrast-enhanced scanning, there was uneven enhancement and a clear boundary, and there was no obvious blood vessel flow void around or inside the tumour.

The possibility of an optic nerve tumour was considered, but the nature of the tumour could not be determined. Given that the patient still had partial remnant eyesight and the high possibility that an operation could cost her eyesight, the usual practice would be to keep the patient under close observation without surgical treatment. However, the authors noted that the left optic nerve of the patient was thickened and oedematous, extending along the optic nerve to the optic chiasma and affecting left visual radiation. In addition, signs of oedema were also present in both frontal lobes. *As a cause of the formation of frontal lobe oedema, we hypothesised that the tumour invaded the optic nerve cells, causing them to produce tumour necrosis factor (TNF). TNF can lead to the cerebral oedema* [10]*. However, we do not have any solid evidence for this.*

In this case study, the tumour not only caused oedema of the optic chiasma and surrounding tissue but also involved the bilateral frontal lobes. There is reason to believe that if the tumour was not removed, the oedema may have further affected the right optic nerve and led to the loss of right visual acuity. Therefore, to prevent the tumour from further affecting the opposite eye and to prevent intracranial lesions, the patient underwent surgical treatment. In addition, MRI examination of the patient in the third month after the surgery indicated that the signal indicating bilateral optic tract and frontal lobe oedema had disappeared. This phenomenon further indicates that these changes were not caused by tumour growth and tumour metastasis, but were reversible. The operation cut off the left optic nerve such that the patient could not restore left visual acuity.

To our knowledge, this is the first reported case of optic nerve HBL with bilateral frontal lobe oedema.

## Data Availability

All data and materials supporting our findings are contained within this manuscript.

## Abbreviations

VHL: Von Hippel-Lindau
HBLs: Hemangioblastomas
VF: Visual field
VEP: Visual evoked potential
MRI: Magnetic resonance imaging
FLAIR: Fluid-attenuated inversion recovery
TNF: Tumour necrosis factor

## References

[1] McGrath LA, Mudhar HS, Salvi SM (2019). Hemangioblastoma of the optic nerve. Surv Ophthalmol.

[2] Singh AD, Shields CL, Shields JA (2001). von Hippel-Lindau disease. Surv Ophthalmol.

[3] Fard MA, Hassanpoor N, Parsa R (2014). Bilateral optic nerve head Angiomas and Retrobulbar Haemangioblastomas in von Hippel-Lindau disease. Neuroophthalmology..

[4] Zheng K, Wu G, Zhang HS, Bao XH, Zhou X, Huang FP (2007). Diagnosis and therapy of hemangioblastoma of optic nerve. Chin J Neuromedicine.

[5] Chen S, Chew EY, Chan CC (2015). Pathology characteristics of ocular von Hippel-Lindau disease with neovascularization of the iris and cornea: a case report. J Med Case Rep.

[6] Wu X, Chen L, Zhang Y, Xie H, Xue M, Wang Y (2018). A novel mutation in the VHL gene in a Chinese family with von Hippel-Lindau disease. BMC Med Genet.

[7] Prabhu K, Daniel RT, Chacko G, Chacko AG (2009). Optic nerve haemangioblastoma mimicking a planum sphenoidale meningioma. Br J Neurosurg.

[8] Turel MK, Kucharczyk W, Gentili F (2017). Optic nerve Hemangioblastomas?A review of visual outcomes. Turk Neurosurg.

[9] Staub BN, Livingston AD, Chevez-Barrios P, Baskin DS (2014). Hemangioblastoma of the optic nerve producing bilateral optic tract edema in a patient with von Hippel-Lindau disease. Surg Neurol Int.

[10] Wu NZ, Ma HP, Wang N (2016). Progression on molecular mechanisms of cerebral edema [J]. Med J Beijing Mil Region.

